# Do Informal Social Ties and Local Festival Participation Relate to Subjective Well-Being?

**DOI:** 10.3390/ijerph18010016

**Published:** 2020-12-22

**Authors:** Young-joo Ahn

**Affiliations:** The Tourism Industry Data Analytics Labs (TIDAL), The College of Hospitality and Tourism Management, Sejong University, Seoul 05006, Korea; yjahn@sejong.ac.kr

**Keywords:** social capital, informal social ties, local festival, subjective well-being, happiness, life satisfaction

## Abstract

The present study examined the relationship between social capital, local festival participation, and subjective well-being. Moreover, this study examined whether the effect of social capital on subjective well-being can be mediated by festival participation. In addition, it examined the decomposition effect of festival participation and control of models for demographic characteristics. Data used are from the International Comparative Survey on Lifestyle and Values (ICSLV) SWB South Korea Survey. The total number of respondents for the analysis is 1694. The findings indicate that trustful relationships with family and relatives, friends, and neighbors are considerably related to subjective well-being than structural social capital. Moreover, the trust of informal social ties shows considerable potential in facilitating individuals’ local festival participation, which is associated with subjective well-being. Individuals who often participate in traditional local festivals in their communities show higher subjective well-being than those who never attend any festivals. Local festivals in communities can play an important role in strengthening links with individuals in these communities and affect community residents’ well-being. Lastly, the findings can suggest beneficial theoretical and practical implications, and enrich the previous literature on social capital and festival participation.

## 1. Introduction

The World Bank [1] reported that the gross domestic product (GDP) in 2018 was $USD 1.619 trillion. South Korea ranked 12th in GDP rankings in 2018. For comparison, South Korea’s GDP per capita was $USD 80.2 in 1960 and $USD 33,433.6 in 2018 [2]. Although previous literature has indicated that GDP reflects the quality of economic growth, it does not show the quality of growth and balanced distribution of wealth and subjective well-being [3]. Moreover, the gap between the poor and rich has been widening, and social issues related to socioeconomic inequality have remained unsolved [4].

The World Happiness Report [5] provided an alternative indicator to understand well-being and happiness, and presented the national happiness of 156 countries. The World Happiness Report [5] indicated that Finland ranked first, followed by Denmark and Norway, people were asked to evaluate quality of life on a scale ranging from 0 to 10. South Korea ranked 54th out of 156 countries, with an actual average life evaluation of 5.895 out of 10 points. The Organization for Economic Cooperation and Development [6] reported that South Korea shows strengths and weaknesses in the well-being dimensions compared with other OECD countries’ average score. Strong well-being dimensions, which show higher average scores than other OECD countries include education and skills, life expectancy, and long-term unemployment. By contrast, low-level well-being dimensions include life satisfaction, social support/social connections, perceived health, job strain, and air quality. People who have relatives or friends they can rely on and acquire social support when they need help show low average scores than those from other OECD countries [6]. In this regard, many studies have highlighted emerging social issues and linked social problems with essential solutions such as social capital, well-being, and community actions for health and quality of life [7,8,9].

People are social animals and exert effort to build social networks and trust with family members and other people [10,11]. These social relationships can be trust-based social assets to develop social bonds or coproduction [11]. Therefore, social capital has been developed and conceptualized with multiple dimensions such as structural and cognitive social capital within formal and informal social groups [11,12]. In particular, informal social ties among family, friends, neighbors, and community social networks can provide various resources (e.g., financial resources, information, and emotional support) [13,14]. For example, when such disasters as earthquakes, and tsunamis, strike communities, disaster research has indicated that informal ties and geographically close neighbors immediately assist other people who ask for help [13].

Subjective well-being can be enhanced through social capital that deepens strong social relationships [15]. Furthermore, it is enhanced through community engagement such as religion, sports and leisure participation, and community campaign involvement [3,15,16]. Previous research has indicated that community social networks increase social support and collective action and facilitate community engagement and productive activities in communities [13,17].

One example of community engagement is a local community festival [18,19,20,21,22]. Meanwhile, a handful of researchers explored the relationship between social capital and festival experiences and only focused on festival participants [18,19,20,21,22,23,24]. Moreover, previous research on social capital and informal social ties among family, friends, and neighbors at the individual level has rarely explored the relationship between social capital, festival participation, and subjective well-being.

In this regard, the purpose of this study was to examine the influence of social capital and festival participation on subjective well-being. Moreover, this study examined whether the effect of social capital on subjective well-being can be mediated by festival participation. This study likewise examined the decomposition effect of festival participation and controlled the proposed models for demographic characteristics.

## 2. Literature Review

### 2.1. Social Capital

Social capital is a complex phenomenon and is used to understand the capacity of individuals’ social structures, social networks, shared norms, and trust that increase collective actions [10,11,25]. Putnam [11] defined social capital as “features of social organization, such as networks, norms, and trust that facilitate coordination and cooperation for mutual benefit (pp. 35–36).” Moreover, social capital is defined and conceptualized in different ways [10,12,26,27,28]. Social capital can be embodied as a social construct among informal social relationships such as family, friends, and neighbors [14]. It can be created, enhanced, or ceased [10]. Social capital’s core components include shared value, norm, reciprocity, and trustworthiness [12].

Common distinct dimensions of social capital are structural and cognitive dimensions. Structural social capital describes observable social networks whereas cognitive social capital reflects trust and reciprocity norms [29]. Social capital is categorized into different types, such as bridging and bonding social capitals [10,26]. Bridging social capital refers to connected relations across lines of individuals who do not have the same social identity whereas bonding social capital reflects strong ties among individuals within each homogeneous group sharing similar characteristics, such as socio-demographic characteristics or social groups [10,26,29,30].

Empirical evidence has shown the association between social capital and positive outcomes such as low suicide and low crime rates and better health condition [29]. Moreover, social networks and reliance on social groups in which individuals belong are associated with increased life satisfaction, physical and mental health, and subjective well-being [30,31,32].

Social capital consists of formal and informal social relations. The extant research has indicated that individuals who have more instrumental and emotional supports from their spouse, family, friends, and neighbors shows lower mortality rates than their counterparts who receive minimal support [33]. Moreover, informal ties and geographically close neighbors are likely to aid other people who need help in difficult situations [13]. Individual and community social networks provide various resources, such as financial resources, information, and emotional support [13,23]. Previous research has likewise demonstrated that people are influenced by social trust in legal policies, firms, or governments in the socio-ecological system [25].

### 2.2. Informal Social Capital and Subjective Well-Being

Social capital has become the focus of numerous studies because it is positively associated with subjective well-being [14,15,34]. The research on subjective well-being has measured the concept by using individuals’ perceived happiness and life satisfaction [3,15,35]. Moreover, previous studies on social capital and subjective well-being have examined the subdimensions of social capital (e.g., structural social capital and cognitive social capital) and subjective well-being [14,15,31,34]. These studies have demonstrated that structural and cognitive social capital positively influences subjective well-being [14]. The sizes of social networks, reliance on social groups, and sense of belonging are associated with increased life satisfaction [31]. High levels of trust make people feel a sense of belonging. Consequently, trust positively links to subjective well-being [17]. 

Previous studies have revealed that the informal social connections’ structural and cognitive social capital influence individuals’ emotions and health conditions [14,29]. In particular, people who have close friends and friendly neighbors show a lower level of negative emotions (e.g., depression). Moreover, people who reported that they have a good relationship with their family, friends, and neighbors are likely to perceive happiness [14]. By contrast, individuals who only have a few interactions with their family and neighbors and trust-based relationships that provide a sense of belonging have been linked to negative physical and mental health conditions [29]. In this regard, the present study focuses on the relationship between individuals’ informal social capital and subjective well-being.

Numerous studies on social capital and subjective well-being have suggested assessing the effects of socio-demographic characteristics and control of these variables [14,16,34,36,37]. Helliwell and Putnam [14] revealed a U-shape curve between age and happiness. The middle-age group showed the lowest level of subjective well-being whereas the young-age group and those over 65 years reported the highest level of subjective well-being. Regarding gender, the results appeared inconsistent, although those living in countries with high-quality governments show higher level of happiness for females than males. People who earn average or higher income showed high reported happiness than those with a low-income level. Education level was positively correlated with subjective well-being, however, education seemed to have no direct effect on subjective well-being. A positive relationship exists between strong religious beliefs and church attendance and subjective well-being [14]. The extant literature in the US and Europe has shown that people who are married, Caucasian, highly educated, and have full-time jobs are likely to report higher happiness; however, age showed a U-shaped curve, which indicates that the middle age group reported lower happiness than the other age groups [14,25]. Therefore, the present study includes five control variables, namely, age, gender, marital status, income, and religion in the proposed models.

### 2.3. Social Capital, Festival Participation, and Subjective Well-Being

Civic engagement in community activities is an influential determinant of life satisfaction [14]. Specifically, civic engagement refers to how residents engage in their communities’ activities [38]. Putnam [11] highlighted that residents living in high-level social capital areas are likely to value community cohesion and commit cooperation for mutual benefits. Social contacts with people in churches and organizations are positively linked with participation in politics [29]. Previous research has indicated that increased social capital can empower residents to engage in local actions [24]. Community social capital networks allow easy access to various beneficial resources for individuals, such as information, aid, and volunteering services [13,39]. Hommerich [17] indicated that trust is developed based on the mutual understanding of social relations, and trust resources increase productive activities and enhance groups’ sense of accomplishment. Trust also contributes to the overall quality of communities.

Local festivals are the collective actions among residents in communities. Positive outcomes of festivals are well documented [40,41]. Local festivals and mega-events held in regions can provide several benefits, such as the host communities’ economic boost, infrastructure improvement, and cultural resource development around festival venues [19,20,40,41,42,43,44]. Moreover, festivals enable local residents and visitors to participate in various activities, thereby increasing their shared value of the local communities and understanding of local cultures [20]. Local residents’ festival participation is essential for increasing support and sustainably retaining local events and festivals in a sustainable manner [23,45]. 

At present, an increasing number of studies have explored the connection among social capital, social actors, and positive and negative outcomes in the contexts of festivals and sports events [18,21,22,23,44,46,47,48,49,50,51,52,53,54,55]. In terms of positive outcomes of festivals and events, previous studies on social capital and festivals have demonstrated that festivals can build community resources, shape social cohesion and social identity, and enhance the quality of life [18,20,56]. Diverse stakeholders are involved in festivals and events and enhance structural and cognitive social capital [22,57]. However, negative perspectives of social capital in festivals and events were also identified [23,24]. Economic, cultural, and social capital may not be equally distributed to all community members and can increase power inequalities [23]. Individuals with lower resources and social capital and vulnerable people would be less likely to engage in festivals and events in communities [24]. Given that social capital is complex, studies on social capital and festivals had explored how social actors build social capital before, during, and after festivals and events and identified the corresponding consequences [24,47]. 

Previous research has indicated that individuals’ existing social capital, social identity, and personal preferences facilitate involvement in festivals and events [21,24,56]. Moreover, cognitive social capital, which is based on trustful relationships, is strongly related to festival engagement and positive outcomes [24,58]. For example, Wilks [24] explored social connection at a music festival site by collecting data from 33 participants. The findings revealed that visitors attended the festival to meet with close relatives and friends and have regular social gatherings at the festival site. Festival attendees occasionally interacted with strangers at the festival or avoided interaction with other festival participants. The results likewise indicated that bonding social capital facilitates festival participation and share social similarities. Furthermore, bonding social capital at festivals appears to be only among people who already knew or have close relationships. Jepson, Stadler, and Spencer [49] explored the relationships between quality of life and community festivals and events and focused on family bonding and collective memory. They conducted several focus group interviews and found several key themes, such as positive memories, family bonding, family connection, and family quality of life. However, previous studies have provided little information on the influence of structural and cognitive social capital of informal social ties on festival participation and subjective well-being, and the effects of festival participation between non-participants and participants in communities on their subjective well-being. Therefore, the present study explored the relationship between social capital, subjective well-being, and festival participation.

## 3. Method

### 3.1. Data Source

This study used the ‘Social Well-being Survey in Asia (SoWSA)’. The survey aims to examine well-being in eight Asian societies (i.e., Indonesia, Japan, Korea, the Philippines, Taiwan, Thailand, Vietnam, and Mongolia) led by Senshu University in Japan. The survey was developed and conducted in seven East and Southeast Asian countries (i.e., Indonesia, Japan, Korea, the Philippines, Taiwan, Thailand, and Vietnam) from 2015 to 2017. The standard survey was developed for social well-being research by Senshu University using a cross-national questionnaire survey.

The present study utilized the data, “International Comparative Survey on Lifestyle and Values (ICSLV) SWB South Korea Survey 2015,” conducted by Seoul National University in South Korea. The nationwide surveys were collected through the website and partially through telephone surveys on 14–22 July 2015. Proportionate quota sampling by sex, age, and region was used for data collection. The total number of respondents was 2000, however, unusable observations with missing values or not were unavailable in this area (e.g., festivals) were removed, leaving 1694 available for analysis.

### 3.2. Measure of Subjective Well-Being

The dependent variables are two measurement items for assessing self-rated subjective well-being, namely, happiness and life satisfaction. Happiness is measured by one question, “How happy are you currently?” on a scale from (0) being ‘very unhappy’ and (10) being ‘very happy’. Life satisfaction is measured by one question, “How satisfied are you currently with your life” on a scale from (0) ‘very unsatisfied’ to (10) ‘very satisfied’.

### 3.3. Measure of Structural Social Capital and Cognitive Social Capital (Trust)

Structural social capital includes four questions about interactions with family and relatives, friends, and neighbors. It also includes three questions about social trust with family and relatives, friends, and neighbors. Social interaction includes three questions (i.e., the interaction of family/relatives, friends, and neighbors). The respondents answer questions, “How often do you interact with the following people? (i.e., family and relatives, friends, and neighbors) on a 5-point Likert scale from (1) not at all, (2) rarely (once a year or every few years), (3) sometimes (once a month, or several times a year), (4) somewhat frequently (once a week, or several times a month), (5) nearly on a daily basis (multiple times per week). The question of the interaction of neighbors was (1) I do not interact with neighbors at all, (2) I have minimal interaction with neighbors, only greeting each other, (3) I have daily interactions and conversations with neighbors, (4) I consult with and share everyday items with some, (5) I feel the same as family with many. Finally, the ratio of neighbors interacted with is measured by a five-point Likert scale from (1) I do not know the names of my neighbors, (2) I only know and interact with my immediate neighbors, (3) I know and interact with about half of my neighbors, (4) I know and interact with many of my neighbors, and (5) I know and interact with most all my neighbors.

Cognitive social capital is measured based on trusting their informal social ties. The respondents answered the question, “To what degree do you feel you can trust or cannot trust the following people? (i.e., family and relatives, friends, neighbors)” on a five-point Likert scale from (1) I cannot trust at all to (5) I can trust a lot.

### 3.4. Measure of Local Festival Participation

In terms of festival participation, the respondents answered the question, “How involved are you in traditional festivals in your area in which many other members of your community participate?” on a five-point Likert scale from (1) never attend, (2) I don’t usually attend, (3) I sometimes participate, (4) I try to participate every time, and (5) I usually participate.

### 3.5. Control Variables

Individual characteristics indicate the age, gender, marital status, household income, occupation, religion, and residential area of the respondents. These variables are considered as important control variables in the subjective well-being literature at the individual level. Age was considered using a continuous variable. Two variables were dummy coded, namely, gender (1 = male, 0 = female) and marital status (1 = married, 0 = others (i.e., single, separated, divorced, widowed)). Respondents’ religion was coded 1 if they have any religious affiliation and 0 otherwise. Household income level (monthly) was grouped into four categories from 1 (less than 2,000,000 KRW (Korean Won)) to 4 (7,000,000 KRW and over).

### 3.6. Statistical Analysis

Demographic characteristics of the respondents are described by using descriptive analysis (see Table 1). Multiple regression models were conducted to investigate the relationship between social capital, festival participation, and subjective well-being. This study conducted a series of mediation analyses using the KHB method [59,60] to test festival participation as a mediating variable between social capital and subjective well-being. The KHB method is conducted to decompose the total effect into a direct effect of the independent variable (i.e., social capital) and the mediator’s indirect effect (i.e., festival participation). The KHB mediation analysis results in confounding ratio, and a confounding percentage. Five control variables were controlled for in the models. Stata 16 was used in this study.

Multiple regression models (i.e., Models 1–10) were conducted. Models 1–6 include structural social capital controlling for demographic characteristics. Cognitive social capital (trust) variables were included in Model 2 and 7. Festival participation (i.e., mediator) was added in Models 4 and 9. Finally, structural and cognitive capitals and festival participation were added in Model 5 and 10 to estimate association with subjective well-being (i.e., happiness, life satisfaction).

## 4. Results

### 4.1. Demographic Characteristics

As shown in Table 1, the descriptive information about the respondents’ demographic characteristics was presented. Approximately 47.52% of the respondents were male. The respondents were almost evenly distributed among the five age groups. The mean age was 43 years old. Approximately 34.83% of the respondents were married. The highest reported household income was between 4,000,000 KRW and less than 7,000,000 KRW (41.26%). Approximately 52% of the respondents reported that they attend religious services.

### 4.2. Social Capital, Festival Participation, and Subjective Well Being

As shown in Table 2, Model 1 indicates that interactions with family and relatives, friends, and neighbors were significantly associated with happiness. Model 5 implied that two structural social capital variables (i.e., interactions with friends and neighbor) were not significantly associated with happiness. However, interactions with family and relatives and ratios of neighbor interactions were significantly associated with happiness in Models 3 and 5. All cognitive social capital variables, family and relatives, friends, and neighbors’ trust were significantly associated with happiness in Models 2, 3, and 5. As shown in Table 3, Model 6 indicated that interactions with family and relatives, friends, and neighbors were significantly associated with life satisfaction. In Model 10, the relationship between the four interaction variables and life satisfaction did not show significant associations. However, trust of family and relatives, friends, and neighbors were all significantly associated with life satisfaction in Models 7, 8, and 10.

### 4.3. Festival Participation and Subjective Well-Being

In Model 4 (see Table 2), individuals who never participated in community festivals were a reference group. Individuals who usually participate in community festivals showed a higher level of happiness than those who never participated. In Model 9 (see Table 3), individuals who never participated in community festivals were a reference group. Individuals who usually participated in community festivals reported higher level of life satisfaction than those who never participated. This included festival participation, perceiving more happiness and life satisfaction; that is, community festival participation appears to be associated with subjective well-being.

### 4.4. Social Capital and Subjective Well-Being Mediated by Festival Participation

The KHB method (Breen, Karlson, & Holm, 2013; Karlson, Holm, & Breen, 2011) was used to report the direct, indirect, and total effects, controlling for control variables (see Table 4 and Table 5). Festival participation significantly mediated the association between the five social capital variables and happiness while controlling for five control variables (i.e., age, gender, marital status, religion, and income). As shown in Table 4, the ratio of neighbor interactions showed the largest mediating effect. The total effect of interaction with family and relatives was 1.23 times larger than the direct effect, and 18.58% of the total effect was due to the mediator variable (i.e., festival participation). The total the effect of ratio of neighbor interaction was 1.48 times larger than the direct effect, and 32.61% of the total effect was due to the mediator variable (i.e., festival participation). The decomposition analysis indicated that the ratio of interaction with neighbors showed the mediating effect. The total effects of ratios of trust of family and relatives, friends, and neighbors were approximately 1.08–1.14 times larger than the direct effect, and about 7.71–12.18% of the total effect was due to the mediator variable (i.e., festival participation).

As shown in Table 5, festival participation significantly mediated the association between three social capital variables and life satisfaction while controlling for five confounding variables (i.e., age, gender, marital status, religion, and income). The total effect of the ratio of trust of family and relatives, friends, and neighbors were approximately 1.08–1.15 times larger than the direct effect, and about 7.28–12.96% of the total effect was due to the mediator variable (i.e., festival participation). This study estimated variance inflation factors (VIF) from the results of the ordinary least-squares (OLS) regression models on all the predictors. The results were found to be greater than one, ranking from the highest (2.56) to the lowest (1.05).

## 5. Discussion

This study aimed to examine whether social capital is associated with subjective well-being at the individual level. A secondary goal was to explore festival participation’s mediation role as civic engagement between social capital and subjective well-being. Several theoretical implications are presented in this study. First, the results indicate that social capital and festival participation are positively associated with subjective well-being (i.e., perceived happiness and life satisfaction). The findings indicate that trustful relationships with family and relatives, friends, and neighbors are more strongly associated with festival participation and subjective well-being than structural social capital. Moreover, bonding social capital is strongly related to festival participation, consistent with Wilks’ [24] finding. The present study’s findings can expand knowledge to understand the relationships among social capital, festival participation, and subjective well-being. The present study reveals that informal social ties’ trust shows considerable potential in facilitating individuals’ local festival participation, associated with subjective well-being.

Second, the findings indicate the effects of demographic characteristics on subjective well-being by using five control variables: Age, gender, marital status, household income, and religion. Middle-aged groups show a lower level of subjective well-being, and the relationship between age and subjective well-being show a U-shape curve. These results are consistent with those previous research [14,34]. Variations in terms of gender, marital status, household income, and religion are also consistent with those of the extant literature [14,34]. The study’s findings reveal that females, married, high household income, and having a religion show a high-level subjective well-being.

Lastly, individuals who often participate in traditional local festivals in their communities show higher subjective well-being than those who never attend any festivals in their communities. This study’s findings can enrich the previous literature on social capital and festival participation [20,22,24,46,47,49,50]. The present research can likewise provide empirical evidence of the relationship between social capital, festival participation, and subjective well-being. The findings also indicate that non-participation in local festivals may be associated with a low-level happiness and life satisfaction. The effects of trust with informal social ties (i.e., family and relatives, friends, neighbors) mediated by festival participation positively influences subjective well-being. Furthermore, the findings of this study present the decomposition of the direct and indirect effects between social capital and subjective well-being mediated by festival participation.

This study presents several practical implications. First, one of the important findings is that cognitive social capital (i.e., trust) was positively associated with subjective well-being more than structural social capital. Previous research has suggested that local and central governments can subsidize local community organizations that need to develop community campaigns to build trustful relationships among family, friends, and neighbors in the community [3,15]. Thus, regularly holding community campaigns and social activities is essential to build trust at the individual level rather than focus on structural social capital (e.g., increasing the number of sociable people and context of social time).

Second, comparing the results of subjective well-being controlling for the socio-demographic characteristics, is consistent with the extant literature [14,25]. Individuals with low household income appear to perceive lower level of happiness and life satisfaction than advantaged groups. Moreover, these disadvantage groups may lack informal and trustful social ties and experience resource shortage. Practitioners and governments may introduce intervention programs to build social capital and increase the sense of belonging in local communities.

Third, local festivals in communities can play an important role in strengthening links with individuals in communities, thereby affecting the communities’ well-being. Previous research on local festivals [18,19] has indicated that celebrations during festivals can bind community members of a community and create opportunities for deepening relationships among festival visitors, uniting diverse stakeholders, and facilitating a vibrant community culture. Local festivals can be one of the essential attractions that increase tourists’ number at tourism destinations and shape destination image [42,56,61,62] Practitioners and government should also develop new strategies to attract various festival stakeholders, such as community residents, the private sectors, and various organizations near local communities. Accordingly, building partnerships and making stakeholders involved in festival operations can create social capital that leads to active festival participation [19]. These festival engagements can be the important community activities individuals engage in, and contribute to increasing subjective well-being.

## 6. Limitations and Further Research Suggestions

This study has several limitations. This study used a nationwide survey, the findings should not be over-generalized. The data used in this study focused on data collected from the domestic population of South Korea. This study also used the same survey questionnaires and collected data from Japan, South Korea, and Taiwan. Further research could obtain insightful information by exploring a cross-cultural comparison between the three countries. The present study focused on measuring informal social ties (i.e., family and relatives, friends, and neighborhood) at the individual level. Further study may need to examine the relationship between social capital, subjective well-being, and festival participation at the municipal level for a profound understanding of the collective nature of Asian countries. Finally, the cross-sectional design may not suggest powerful empirical evidence of differences in subjective well-being over time. Hence, further research using longitudinal data would be required to identify the strong determinants of the relationships among social capital, festival participation, and subjective well-being.

## Figures and Tables

**Table 1:** Descriptive statistics for the final sample.

Variable	*N* (%), Mean (SD)	Range
Individual characteristics (*n* = 1694)		
Happiness (Mean, SD)	6.014 (2.036)	1–10
Life satisfaction	5.787 (2.053)	1–10
Age	43 (12.17)	20–69
Gender		
Male	805 (47.52)	Dummy (1)
Female	889 (52.48)	Dummy (0)
Marital status		
Married	590 (34.83)	Dummy (1)
Others (single, divorced, widowed)	1104 (65.17)	Dummy (0)
(Monthly) Household income		
(1) Less than 2,000,000 KRW	148 (8.74)	
(2) 2,000,000–less than 4,000,000 KRW	511 (30.17)	
(3) 4,000,000–less than 7,000,000 KRW	699 (41.26)	
(4) 7,000,000 and over KRW	336 (19.83)	
Religion		
No	0.479 (0.500)	Dummy (0)
Yes	0.521 (0.500)	Dummy (1)
Structural social capital (interaction)		
Family and relatives	2.712 (0.771)	1–5
Friends	3.223 (0.786)	1–5
Neighbors	2.723 (1.110)	1–5
Ratio of interaction with Neighbors	2.253 (1.116)	1–5
Cognitive social capital (trust)		
Family and relatives	3.684 (0.829)	1–5
Friends	3.361 (0.738)	1–5
Neighbors	2.825 (0.734)	1–5
Festival participation	2.607 (0.961)	1–5
(1) I never attend	227 (13.40)	
(2) I don’t usually attend	526 (31.05)	
(3) I sometimes participate	663 (39.14)	
(4) I try to participate every time	241 (14.23)	
(5) I usually participate	37 (2.18)	

USD1 is approximately equivalent to 1209 KRW (Korean Won).

**Table 2:** Influences of social capital and festival participation on happiness.

	Model 1	Model 2	Model 3	Model 4	Model 5
Age	−0.015 *** (0.005)	−0.015 *** (0.004)	−0.016 *** (0.004)	−0.013 *** (0.005)	−0.015 *** (0.004)
Age^2^	0.001 ** (0.000)	0.001 *** (0.000)	0.001 ** (0.000)	0.001 *** (0.000)	0.001 ** (0.000)
Gender	−0.248 ** (0.092)	−0.376 *** (0.091)	−0.390 *** (0.090)	−0.240 ** (0.093)	−0.395 *** (0.090)
Married	0.544 *** (0.122)	0.552 *** (0.118)	0.486 *** (0.118)	0.528 *** (0.123)	0.424 *** (0.118)
Religion	0.390 *** (0.094)	0.396 *** (0.092)	0.345 *** (0.091)	0.417 *** (0.094)	0.322 *** (0.090)
Income	0.408 *** (0.054)	0.403 *** (0.053)	0.3725 *** (0.0525)	0.439 *** (0.054)	0.362 *** (0.052)
Interaction (relatives)	0.298 *** (0.065)	-	0.160 * (0.064)	-	0.149 * (0.064)
Interaction (friends)	0.214 *** (0.063)	-	0.056 (0.063)	-	0.024 (0.063)
Interaction (neighbors)	0.149 ** (0.056)	-	0.092 (0.055)	-	0.053 (0.055)
Ratios of neighbor interaction	0.179 ** (0.056)	-	0.171 ** (0.054)	-	0.113 * (0.055)
Trust (family)	-	0.394 *** (0.066)	0.377 *** (0.066)	-	0.363 *** (0.066)
Trust (friends)	-	0.196 * (0.079)	0.192 * (0.079)	-	0.198 * (0.079)
Trust (neighbors)	-	0.446 *** (0.076)	0.273 *** (0.079)	-	0.266 *** (0.079)
Festival participation 1	-	-	-	0.000 (.)	0.000 (.)
Festival participation 2	-	-		0.504 *** (0.151)	0.372 * (0.145)
Festival participation 3	-	-	-	0.989 *** (0.146)	0.708 *** (0.143)
Festival participation 4	-	-	-	1.429 *** (0.177)	0.957 *** (0.179)
Festival participation 5	-	-	-	1.952 *** (0.339)	1.103 ** (0.336)
Constant	4.560 *** (0.668)	3.658 *** (0.651)	2.973 *** (0.666)	6.055 *** (0.633)	2.786 *** (0.665)
Sample	1694	1694	1694	1694	1694
R-square	0.157	0.189	0.212	0.141	0.230
Adjusted R-square	0.152	0.185	0.206	0.136	0.222

* *p* < 0.05, ** *p* < 0.01, *** *p* < 0.001.

**Table 3:** Influences of social capital and festival participation on life satisfaction.

	Model 6	Model 7	Model 8	Model 9	Model 10
Age	−0.016 *** (0.005)	−0.016 *** (0.005)	−0.016 * (0.004)	−0.015 *** (0.005)	−0.015 ** (0.004)
Age^2^	0.001 ** (0.000)	0.001 *** (0.000)	0.001 ** (0.000)	0.001 ** (0.000)	0.001 ** (0.000)
Gender	−0.158 (0.093)	−0.299** (0.091)	−0.309*** (0.091)	−0.153 (0.093)	−0.312 *** (0.090)
Married	0.633 *** (0.123)	0.622 *** (0.119)	0.572 *** (0.119)	0.596 *** (0.123)	0.506 *** (0.118)
Religion	0.368 *** (0.095)	0.366 *** (0.092)	0.322 *** (0.092)	0.384 *** (0.095)	0.298 ** (0.091)
Income	0.472 *** (0.054)	0.458 *** (0.053)	0.431 *** (0.053)	0.500 *** (0.054)	0.421 *** (0.052)
Interaction (relatives)	0.259 *** (0.066)	-	0.117 (0.065)	-	0.104 (0.064)
Interaction (friends)	0.250 *** (0.063)	-	0.080 (0.063)	-	0.048 (0.063)
Interaction (neighbors)	0.150 ** (0.057)	-	0.098 (0.056)	-	0.057 (0.055)
Ratios of neighbor interaction	0.130 * (0.056)	-	0.127 * (0.055)	-	0.064 (0.055)
Trust (family)	-	0.433 *** (0.066)	0.418 *** (0.067)	-	0.402 *** (0.066)
Trust (friends)	-	0.251 ** (0.079)	0.240 ** (0.080)	-	0.244 ** (0.079)
Trust (neighbors)	-	0.357 *** (0.076)	0.209 ** (0.080)	-	0.208 ** (0.079)
Festival participation 1	-	-	-	0.000 (.)	0.000 (.)
Festival participation 2	-	-	-	0.461 ** (0.151)	0.349 * (0.145)
Festival participation 3	-	-	-	0.974 *** (0.147)	0.722 *** (0.144)
Festival participation 4	-	-	-	1.448 *** (0.178)	1.044 *** (0.179)
Festival participation 5	-	-	-	1.705 *** (0.340)	0.978 ** (0.338)
Constant	4.089 *** (0.673)	3.002 *** (0.651)	2.366 *** (0.669)	5.545 *** (0.634)	2.178 ** (0.667)
Sample	1694	1694	1694	1694	1694
R-square	0.157	0.201	0.217	0.151	0.238
Adjusted R-square	0.152	0.197	0.211	0.146	0.230

* *p* < 0.05, ** *p* < 0.01, *** *p* < 0.001.

**Table 4:** Regression for effects for the association between social capital and happiness, mediated by festival participation.

	Social Capital Interaction with Family/ Relatives	Social Capital Ratio of Neighbor Interaction	Social Capital Trust of Family/Relatives	Social Capital Trust of Friends	Social Capital Trust of Neighbors
*n*	1694	1694	1694	1694	1694
Total effect	0.4881 *** (0.0597)	0.3561 *** (0.0422)	0.6527 *** (0.0550)	0.6594 *** (0.0611)	0.7417 *** (0.0626)
Direct effect	0.3974 *** (0.0907)	0.2400 *** (0.0450)	0.6020 *** (0.0553)	0.6085 *** (0.0613)	0.6514 *** (0.0636)
Indirect effect	0.0907 *** (0.0164)	0.1161 *** (0.0174)	0.0506 *** (0.0133)	0.0509 ** (0.0147)	0.0903 *** (0.0166)
Confounding ratio	1.2282	1.4839	1.0841	1.0836	1.1387
Confounding percentage	18.58	32.61	7.76	7.71	12.18
R square	0.16	0.16	0.20	0.19	0.19

** *p* < 0.01, *** *p* < 0.001.

**Table 5:** Regression for effects for the association between social capital and life satisfaction, mediated by festival participation.

	Social Capital Trust of Family/Relatives	Social Capital Trust of Friends	Social Capital Trust of Neighbors
*n*	1694	1694	1694
Total effect	0.6846 *** (0.0550)	0.6899 *** (0.0610)	0.7011 *** (0.0630)
Direct effect	0.6347 *** (0.0552)	0.6397 *** (0.0613)	0.6103 *** (0.0640)
Indirect effect	0.0499 *** (0.0131)	0.0502 ** (0.0145)	0.0909 *** (0.0167)
Confounding ratio	1.0787	1.1489	1.0785
Confounding percentage	7.29	12.96	7.28
R square	0.21	0.19	0.20

** *p* < 0.01, *** *p* < 0.001.

## Data Availability

The data presented in this study are available on request from the corresponding author.
